# Synthetic cathinone (α-pyrrolidinohexanophenone): an emerging threat

**DOI:** 10.1192/j.eurpsy.2022.1193

**Published:** 2022-09-01

**Authors:** V. Rosello-Molina, A. Hervas Aparisi, M. Simon Blanes, J. Tejera Nuñez, E. Moreno Soldado, I. Meri Abad, O. Sparano Ros, C. Ribera, M. Esteban, T. De Vicente Muñoz

**Affiliations:** Hospital La Ribera, Psychiatry, Alzira, Spain

**Keywords:** drugs, α-pyrrolidinohexanophenone, dual disorders, Psychosis

## Abstract

**Introduction:**

Alpha-pyrrolidinohexanophenone (α-PHP) is a synthetic cathinone with uneven distribution throughout the world. Its use is not uniformly regulated and its distribution is legal in some European countries. Easily accessible and available through different websites. Synthetic cathinones inhibit monoamine transporters which include dopamine, norepinephrine, and serotonin, resulting in increased neurotransmitter synaptic concentration. Ways of administration show wide range regarding latency period. Onset and appearance of symptoms as well as their duration and intensity may fluctuate. A decreasing order of latency (oral, inhaled, sublingual and intravenous) has been reported. α-PHP can result in the appearance of psychiatric symptoms, include among others, intoxication with sensory perception disturbances and α-PHP -induced psychotic episodes.

**Objectives:**

The aim of our study was to assess the epidemiology, clinical and legal features regarding Alpha-pyrrolidinohexanophenone (α-PHP).

**Methods:**

Review the current bibliography to upgrade the existing knowledge. -Present assorted cases with diverse clinical features. All cases include variability through psychopathological interview, symptoms assessment and treatment response according to rating scales (PANSS, YMRS). -Evaluate different treatment administration ways during acute phase and after hospital discharge.

**Results:**

Differences were observed after hospitalization in the response using diverse rating scales. We used antipsychotics to treat intoxication with sensory perception disturbances and α-PHP -induced psychotic episodes. α-PHP had a negative impact on the quality of life of the patients.

**Conclusions:**

α-PHP is a synthetic cathinone with potential risk to mental health and life of users. It is mandatory to implement common legislation all through the European Union to prevent its use and possible implications on population’s mental health.

**Disclosure:**

No significant relationships.

